# Histamine in the brain

**DOI:** 10.3389/fnsys.2014.00064

**Published:** 2014-04-28

**Authors:** M. Beatrice Passani, Pertti Panula, Jian-Sheng Lin

**Affiliations:** ^1^NEUROFARBA, Universitá di FirenzeFirenze, Italy; ^2^Neuroscience Center and Institute of Biomedicine, University of HelsinkiHelsinki, Finland; ^3^Centre de Recherche en Neurosciences de Lyon, Université Claude-Bernard LyonLyon, France

**Keywords:** histamine receptors, cognition, wakefulness, heterogeneity, anxiety

Brain histamine promotes wakefulness and orchestrates disparate behaviors and homeostatic functions. Recent evidence suggests that aberrant histamine signaling in the brain may also be a key factor in addictive behaviors and degenerative disease such as Parkinson's diseases and multiple sclerosis. The intent of this research Topic is to provide an overview of the recent advances in the understanding of the many functions of brain histamine and to propose neurobiological substrates and mechanisms of action that might explain the reasons why the histaminergic system is a potential target for therapeutic interventions. This may justify the search for new histaminergic compounds.

The authors that contributed to this e-book offered several approaches to the study of brain histamine function. Tomasch et al. (2012) synthesized a novel fluorescent ligand of the human histamine H3 receptor with potential to be used as pharmacological tools for visualization in different tissues. Shibuya et al. (2012) by using positron emission tomography (PET) in the human brain examined whether the levels of neuronal release of histamine might change binding of [(11)C]doxepin to the H1 receptors (a standard method for measuring H1 distribution) under the influence of physiological stimuli.

Histamine acts as a modulator of several neurotransmitters in the brain and its role in promoting wakefulness has for long overshadowed other important functions. In fact, histamine signaling controls feeding behavior in a complex fashion and it has been considered for long a satiety system as brain histamine decreases the drive to consume food. In their paper, Ishizuka and Yamatodani (2012) demonstrated the fine regulation of histamine release during feeding and in taste perception. Furthermore, they showed that histamine neurons respond to both mechanical and chemical sensory input from the oral cavity, as may be expected for a danger detection system.

Brain histamine is crucial for motivation and goal-directed behaviors as reviewed by Torrealba et al. (2012). The authors evaluated recent works demonstrating that histamine is differentially involved in the appetitive, food anticipatory responses, and in food consumption, suggesting that it may have an important role in abnormal appetites not only for food but also for substances of abuse. Indeed, preclinical studies on both rats and mice are hinting at a possible role of the histaminergic system in alcohol consumption, as blockade of the H_3_ receptor (which regulates histamine and other neurotransmitters' release), decreases alcohol drinking in several behavioral tasks, like operant alcohol administration and “drinking in the dark” paradigm (Nuutinen et al., 2012). However, the authors caution that despite the evidence that the H_3_ receptor is a key element in alcohol drinking and place preference, the role of histamine in these behaviors is poorly understood and deserves further investigation.

The importance of H_3_ receptor signaling in the brain to acquire and store short- and long- term memories has been documented extensively. However a limited number of studies have investigated the role of the H_3_ receptor in anxiety. By using novel behavioral test, Abuhamdah et al. (2012) present their results with selective agonist and antagonist for the H_3_ receptor providing new evidence that the H_3_R may have a role in fear-induced avoidance responses, but not in anxiety. In addition, Vohora and Bhowmik (2012) provided comprehensive neurobiological/neurochemical evidence of the role of histaminergic H3 receptor antagonists in the physiopathology of cognitive dysfunction and motor impairments.

Dysfunctions of the histaminergic system may also contribute to the pathogenesis of multiple sclerosis and its murine model of experimental autoimmune encephalomyelitis, although the role of the different histamine receptors is complex and still controversial (Passani and Ballerini, 2012).

Histaminergic neurons are sensitive to CO_2_, Yanovsky et al. (2012) showed the complex mechanism of histaminergic neuron activation by acidification in murine brain slices. Their results contribute to understand the neuronal mechanisms controling acid/CO_2_-induced arousal in hepatic encephalopathy and obstructive sleep apnoea.

Recent evidence summarized by Blandina et al. (2012) suggest that such a complexity of the brain histamine system may be served by different neuronal subpopulations that are recruited at different times during the unfolding of a specific behavior. Histamine neurons send broad projections within the CNS that are organized in functionally distinct circuits impinging on different brain regions. This implies independent functions of subsets of histamine neurons according to their terminal projections and their selective participation in different aspects of behavioral responses.

In conclusion, we believe that this Research Topic offered an inter-disciplinary forum that improved our current knowledge of the role of brain histamine. It also provided the necessary drive to stimulate innovation in clinical practice to manage and treat neurological disorders.

## Conflict of interest statement

The authors declare that the research was conducted in the absence of any commercial or financial relationships that could be construed as a potential conflict of interest.
